# Supplementary material for: Taxonomic Characterization, and Secondary Metabolite Analysis of Streptomyces triticiradicis sp. nov.: A Novel Actinomycete with Antifungal Activity
Source: Microorganisms. 2020 Jan 5;8(1):77. doi: 10.3390/microorganisms8010077 (PMC7023189; doi:10.3390/microorganisms8010077)
Supplement: Supplementary file 1 [file microorganisms-08-00077-s001.pdf]

Supporting Information

Table S1. Cultural characteristics of strain NEAU-H2^T, *S. rhizosphaerihabitans* NBRC 109807^T, *S. populi* A249^T and *S. siamensis* NBRC 108799^T. Abbreviations: BA, Bennett's agar; CA, Czapek's agar; NA, Nutrient agar.

Characteristic	NEAU-H2^T	NBRC 109807^T	A249^T	NBRC 108799^T. 111515532 15532^T
Growth on ISP1				
Aerial mycelium	White	Pinkish White	Pinkish White	White
Substrate mycelium mycelium	White	Pinkish White	Pinkish White	Grayish Greenish Yellow
Growth on ISP2				
Aerial mycelium	Moderate Yellow	Pale Violet	White	White
Substrate mycelium	Moderate Yellow	Pale Violet	White	Medium Gray
Growth on ISP3				
Aerial mycelium	White	Pale Blue	Pale Blue	White
Substrate mycelium	Light Yellow	Pale Blue	Pale Blue	Light Bluish Gray
Growth on ISP4				
Aerial mycelium	Light Yellow	White	White	White
Substrate mycelium	Light Yellow GreenGreenGreen	Pale Yellow	White	White
Growth on ISP5				
Aerial mycelium	Pale Yellow	White	Pale Green	Strong Greenish Yellow
Substrate mycelium	Pale Yellow	White	Pale Green	Strong Greenish Yellow
Growth on ISP6				
Aerial mycelium	Light Olive Gray	Very Pale Blue	Very Pale Blue	White
Substrate mycelium	Light Olive Gray	Very Pale Blue	Very Pale Blue	White
Diffusible pigment	Dark Greenish Olive	None	None	None
Growth on ISP7				
Aerial mycelium	Yellow White	White	White	White
Substrate mycelium	Yellow White	White	White	White
Growth on NA				
Aerial mycelium	Greenish White	Light Greenish Gray	Bluish White	White
Substrate mycelium	Greenish White	Light Greenish Gray	Bluish White	White
Growth on CA				
Aerial mycelium	White	White	White	White
Substrate mycelium	White	White	White	White
Growth on MBA				
Aerial mycelium colour	Pale Greenish Yellow	White	Pale Greenish Yellow	Pale Yellow
Substrate mycelium colour	Pale Greenish Yellow	Pale Greenish Yellow	Pale Greenish Yellow	Pale Yellow

Table S2. MLSA distance values for selected strains of strains NEAU-H2^T in this study. Strains: 1, NEAU-H2^T; 2, *Streptomyces populi* A249^T; 3, *Streptomyces siamensis* NBRC 108799^T; 4, *Streptomyces rhizosphaerihabitans* NBRC 109807^T; 5, *Streptomyces mirabilis* NRRL ISP-5553^T; 6, *Streptomyces scabiei* NRRL B-1652^T; 7, *Streptomyces griseoviridis* NRRL ISP-5229^T; 8, *Streptomyces yaanensis* NRRL B-24964^T; 9, *Streptomyces ciscaucasicus* KCTC 19958^T; 10, *Streptomyces europaeiscabiei* ST1229^T; 11, *Streptomyces spiralis* NRRL B-16922^T; 12, *Streptomyces longisporus* NRRL B-5336^T; 13, *Streptomyces humidus* NRRL B-3172^T; 14, *Streptomyces rishiriensis* NRRL B-3239^T; 15, *Streptomyces stelliscabiei* IBSBF 2085^T; 16, *Streptomyces griseosporeus* NRRL B-12498^T; 17, *Streptomyces fimbriatus* NRRL B-3175^T; 18, *Streptomyces corchorusii* NRRL B-2904^T; 19, *Streptomyces venetus* CMU-AB225^T; 20, *Streptomyces massasporeus* NRRL B-3300^T; 21, *Streptomyces indiaensis* NRRL B-24311^T; 22, *Streptomyces levis* NRRL B-16370^T; 23, *Streptomyces prasinosporus* NRRL B-12431^T; 24, *Streptomyces labedae* NRRL B-5616^T; 25, *Streptomyces glaucescens* NRRL B-2706^T; 26, *Streptomyces lincolnensis* LC-G^T; 27, *Streptomyces avermitilis* NBRC 14893^T; 28, *Streptomyces brasiliensis* NRRL B-3327^T; 29, *Streptomyces aurantiacus* NRRL ISP-5412^T; 30, *Kitasatospora setae* KM-6054^T.

Strain	MLSA (Kimura 2-parameter) distance																											
	1	2	3	4	5	6	7	8	9	10	11	12	13	14	15	16	17	18	19	20	21	22	23	24	25	26	27	28	29
1	-																												
2	0.015																												
3	0.045	0.044																											
4	0.081	0.088	0.088																										
5	0.050	0.051	0.053	0.098																									
6	0.074	0.076	0.081	0.086	0.086																								
7	0.082	0.088	0.089	0.051	0.095	0.083																							
8	0.077	0.080	0.070	0.090	0.079	0.085	0.079																						
9	0.064	0.068	0.068	0.071	0.075	0.074	0.071	0.065																					
10	0.068	0.069	0.067	0.083	0.074	0.084	0.087	0.068	0.058																				
11	0.070	0.070	0.073	0.082	0.083	0.081	0.069	0.061	0.071	0.085																			
12	0.068	0.073	0.070	0.065	0.081	0.076	0.069	0.066	0.053	0.070	0.066																		
13	0.063	0.069	0.063	0.070	0.074	0.068	0.069	0.074	0.057	0.067	0.076	0.055																	
14	0.063	0.069	0.065	0.074	0.074	0.066	0.071	0.075	0.055	0.062	0.073	0.057	0.015																
15	0.075	0.074	0.072	0.084	0.082	0.085	0.084	0.076	0.068	0.050	0.083	0.082	0.073	0.068															
16	0.073	0.079	0.083	0.069	0.089	0.086	0.059	0.067	0.074	0.086	0.063	0.069	0.067	0.065	0.087														
17	0.073	0.072	0.071	0.065	0.089	0.087	0.057	0.068	0.065	0.079	0.064	0.069	0.067	0.067	0.077	0.043													
18	0.066	0.068	0.071	0.068	0.088	0.077	0.056	0.068	0.066	0.077	0.054	0.058	0.063	0.065	0.082	0.053	0.048												
19	0.084	0.088	0.083	0.088	0.082	0.098	0.077	0.087	0.076	0.091	0.086	0.080	0.084	0.083	0.092	0.075	0.068	0.075											
20	0.082	0.087	0.085	0.086	0.089	0.100	0.078	0.087	0.077	0.092	0.081	0.076	0.079	0.074	0.092	0.070	0.069	0.077	0.035										
21	0.085	0.087	0.087	0.084	0.091	0.099	0.075	0.086	0.081	0.092	0.081	0.078	0.080	0.077	0.093	0.067	0.068	0.076	0.035	0.011									
22	0.083	0.088	0.082	0.083	0.088	0.097	0.069	0.083	0.076	0.090	0.069	0.073	0.075	0.077	0.093	0.063	0.063	0.071	0.043	0.034	0.034								
23	0.073	0.079	0.074	0.060	0.089	0.093	0.058	0.071	0.069	0.082	0.064	0.070	0.069	0.073	0.086	0.053	0.031	0.054	0.071	0.073	0.071	0.065							
24	0.071	0.076	0.074	0.073	0.091	0.079	0.058	0.069	0.073	0.084	0.060	0.068	0.062	0.061	0.088	0.057	0.054	0.043	0.080	0.084	0.085	0.077	0.062						
25	0.072	0.076	0.076	0.070	0.084	0.080	0.052	0.070	0.067	0.082	0.061	0.065	0.065	0.069	0.083	0.054	0.051	0.028	0.074	0.076	0.075	0.067	0.055	0.053					
26	0.068	0.071	0.073	0.071	0.084	0.077	0.062	0.072	0.057	0.076	0.069	0.066	0.070	0.066	0.063	0.074	0.073	0.069	0.088	0.085	0.085	0.078	0.077	0.074	0.072				
27	0.051	0.053	0.040	0.087	0.051	0.080	0.090	0.066	0.063	0.063	0.077	0.075	0.064	0.061	0.065	0.083	0.072	0.077	0.082	0.087	0.087	0.089	0.077	0.080	0.078	0.069			
28	0.073	0.075	0.077	0.079	0.083	0.084	0.069	0.071	0.067	0.083	0.063	0.066	0.064	0.062	0.085	0.064	0.056	0.064	0.086	0.079	0.078	0.074	0.060	0.064	0.068	0.073	0.076		
29	0.068	0.070	0.068	0.087	0.076	0.081	0.076	0.080	0.080	0.082	0.073	0.075	0.079	0.077	0.082	0.080	0.079	0.073	0.093	0.097	0.096	0.089	0.081	0.081	0.082	0.083	0.073	0.070	
30	0.159	0.157	0.162	0.156	0.169	0.161	0.158	0.175	0.169	0.174	0.154	0.167	0.156	0.155	0.180	0.156	0.161	0.162	0.177	0.175	0.174	0.171	0.163	0.162	0.156	0.164	0.166	0.172	0.161

Table S3. Genome sequence features of strain NEAU-H2^T and *S. populi* A249^T.

Features	NEAU-H2^T	*S. populi* A249^T
Bioproject	PRJNA574783	PRJNA421064
Accession No.	WBKG00000000	PJOS00000000
Sequencing Technology	Illumina HiSeq	Illumina HiSeq
Assembly method	SOAP denovo v. 2.04	SOAP denovo v. 2.04
Genome coverage	152.0x	310.0x
N50	167,996	75,083
Contigs	135	279
Genome size (bp)	9,921,301	9,587,301
DNA GC content (%)	71.5	71.7
Number of genes	8,929	8,603
Protein coding genes	8,287	7,911
rRNAs	12	7
tRNAs	73	70
ncRNAs	3	3

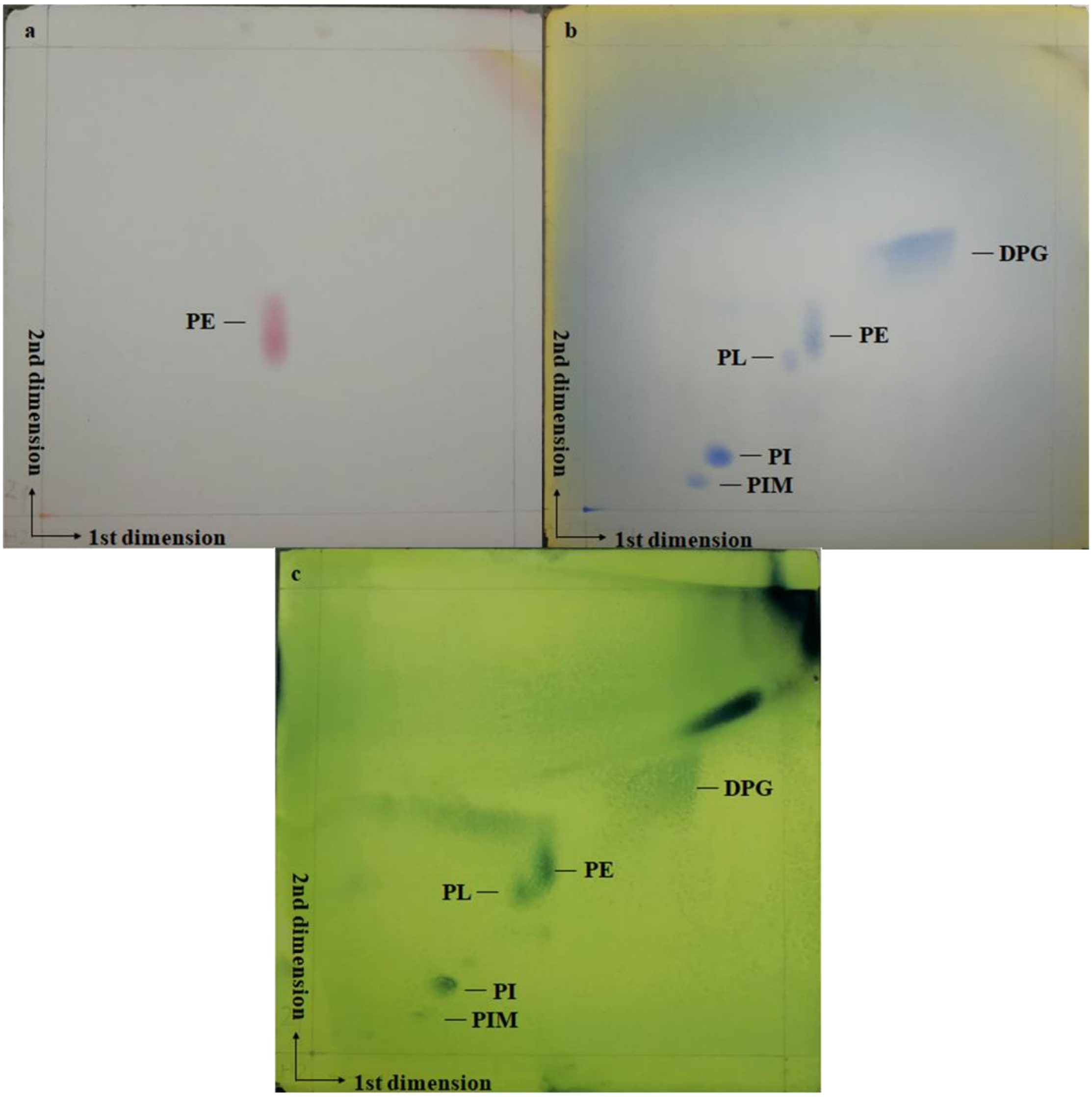

Figure S1. The phospholipids of strain NEAU-H2^T. **a**, Using ninhydrin reagent; **b**, Using molybdenum blue reagent; **c**, Using molybdophosphoric acid reagent. 1st dimension:Chloroform:Methanol:Water (65:25:4, *v*/*v*); 2nd dimension: Chloroform:Acetic acid:Methanol:Water (80:18:12:5, *v*/*v*). Abbreviations: DPG, diphosphatidylglycerol; PE,

phosphatidylethanolamine; PI, phosphatidylinositol; PIM, phosphatidylinositol mannoside; PL, unidentified phospholipid.

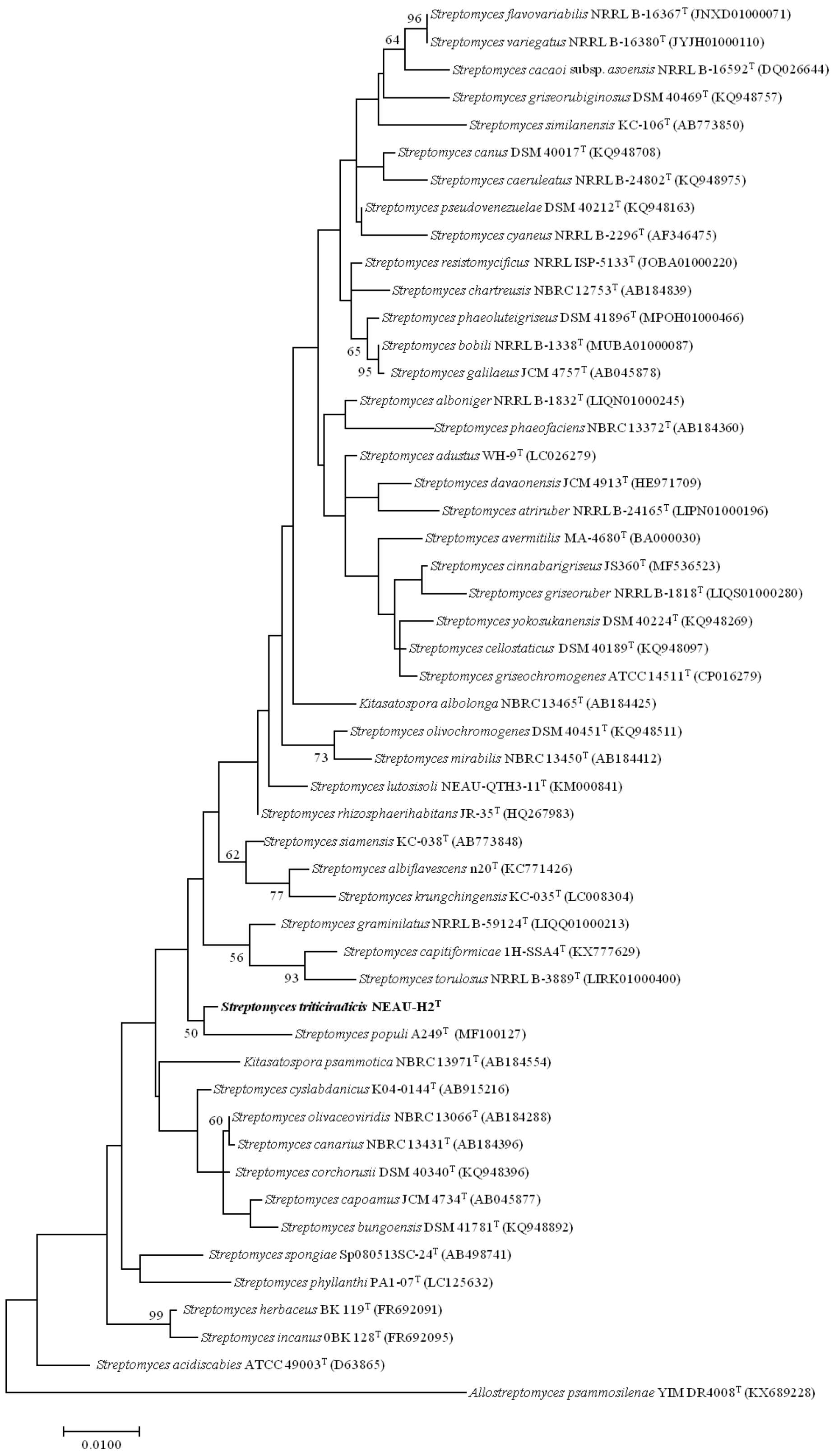

Figure S2. Maximum-likelihood tree based on 16S rRNA gene sequences (1418 bp) showing relationships of NEAU-H2^T (in bold) with related taxa which are the top 50 type strains of *Streptomyces* species of gene sequence similarities based on analysis using EzTaxon-e. Only bootstrap

values above 50% (percentages of 1000 replications) are indicated. *Allostreptomyces psammosilenae* YIM DR4008^T (KX689228) was used as an outgroup. Bar, 0.01 nucleotide substitutions per site.

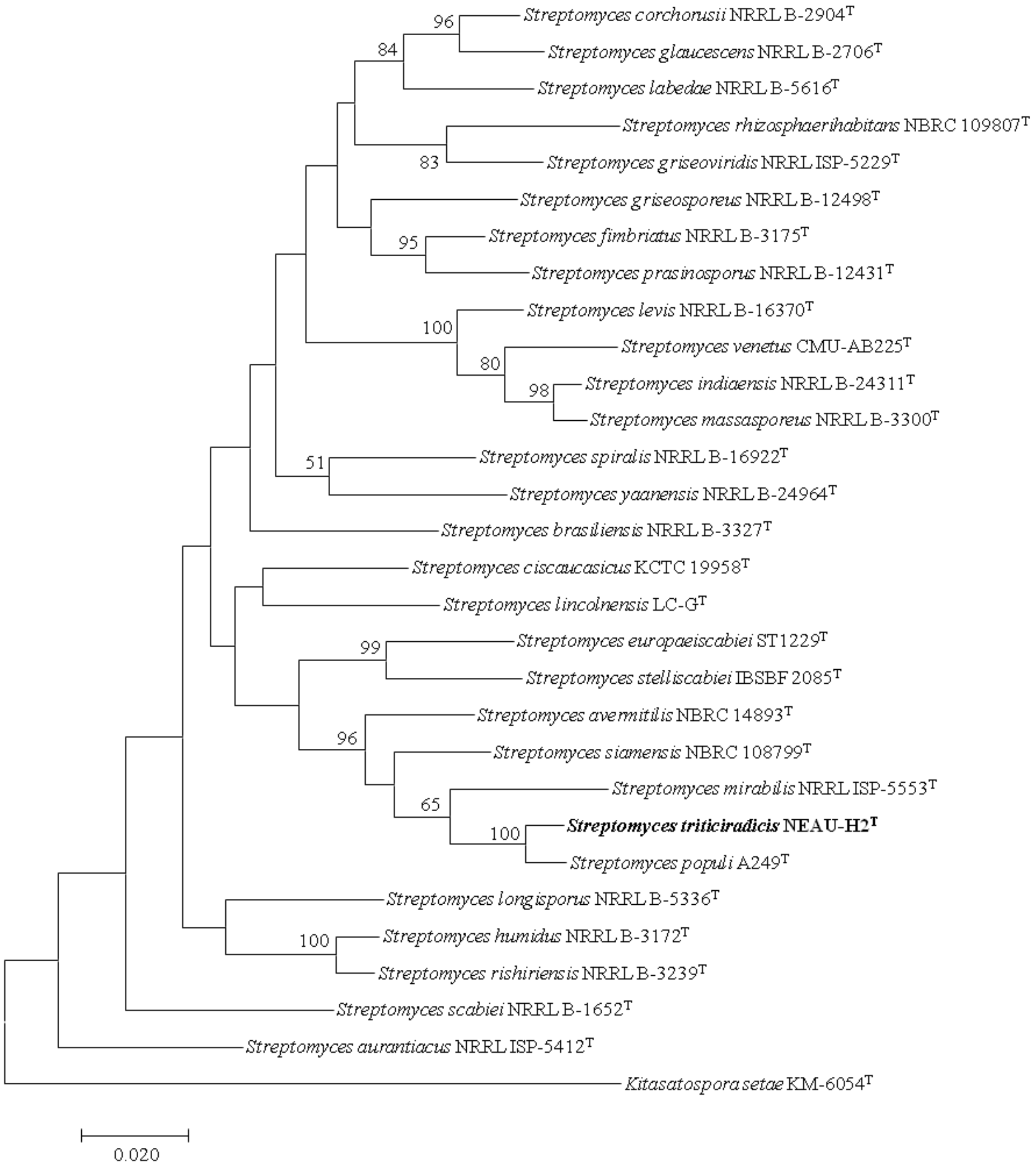

Figure 3. Maximum-likelihood tree based on MLSA analysis of the concatenated partial sequences (2060 bp) from five housekeeping genes (*atp*D, *gyr*B, *rec*A, *rpo*B and *trp*B) of strain NEAU-H2^T (in bold) with related taxa. Only bootstrap values above 50% (percentages of 1000 replications) are indicated. *Kitasatospora setae* KM-6054^T was used as an outgroup. Bar, 0.02 nucleotide substitutions per site.

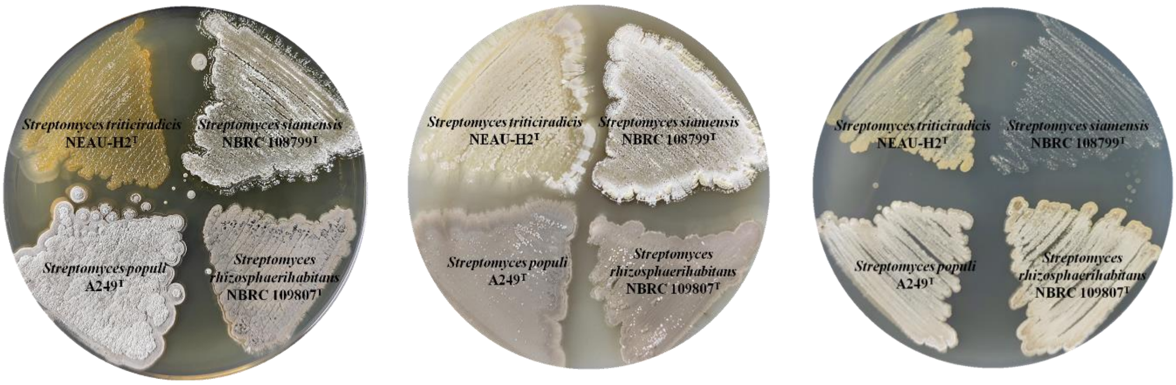

ISP 2 ISP 3 ISP 4

Figure S4. Cultural characteristics of strain NEAU-H2^{T}, *Streptomyces rhizosphaerihabitans* NBRC 109807^{T}, *Streptomyces populi* A249^{T} and *Streptomyces siamensis* NBRC 108799^{T} observed on ISP 2, ISP 3 and ISP 4 media at 28 °C for 2 weeks.

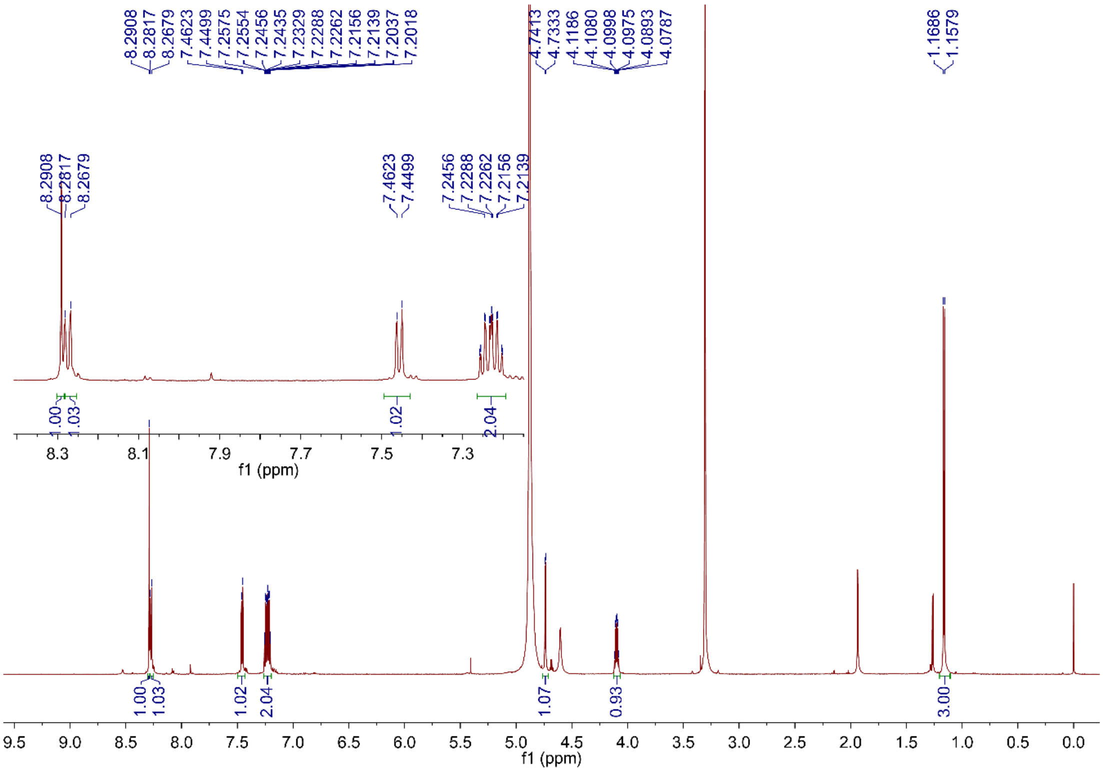

(A)

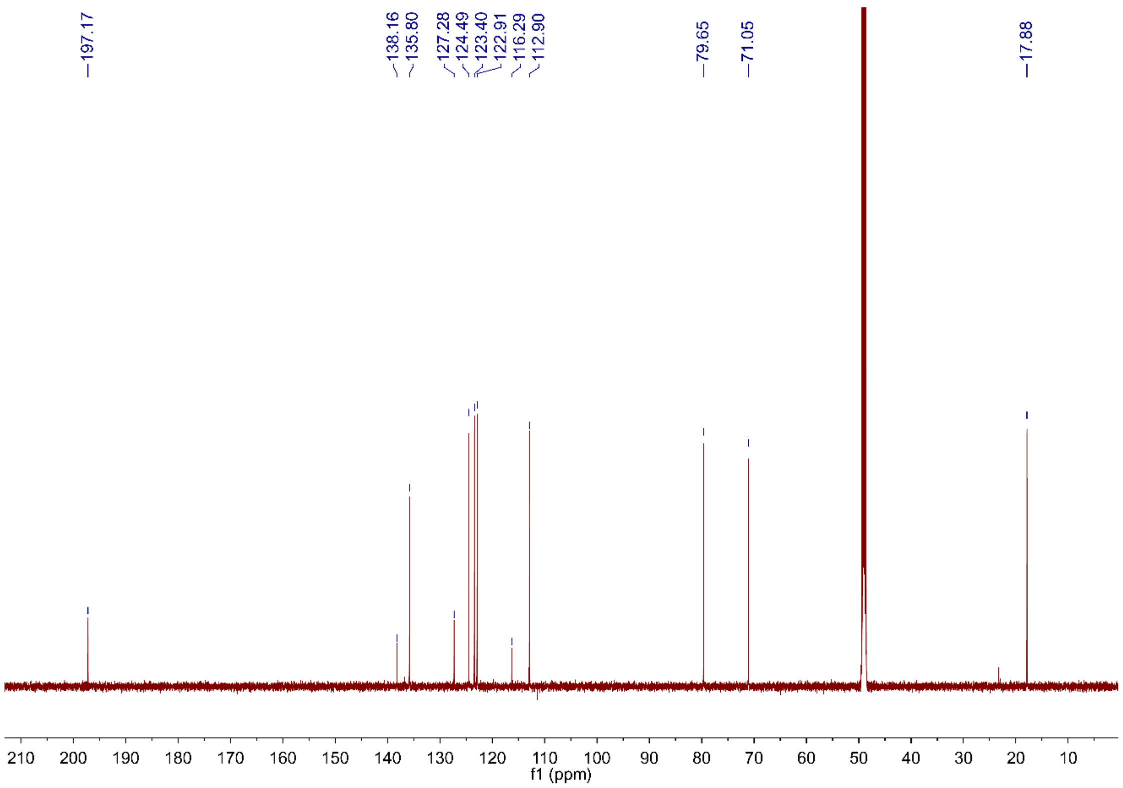
197.17
138.16
135.80
127.28
124.49
123.40
122.91
116.29
112.90
79.65
71.05
17.88
210 200 190 180 170 160 150 140 130 120 110 100 90 80 70 60 50 40 30 20 10
f1 (ppm)

(B)

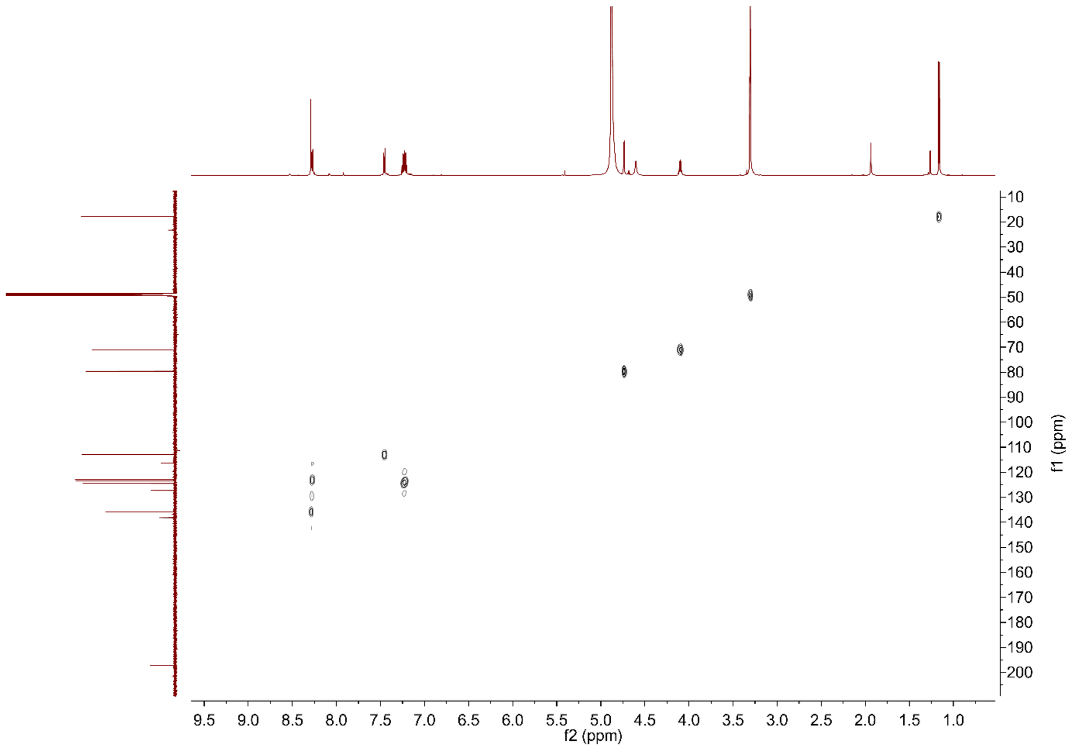
9.5 9.0 8.5 8.0 7.5 7.0 6.5 6.0 5.5 5.0 4.5 4.0 3.5 3.0 2.5 2.0 1.5 1.0
f2 (ppm)
10 20 30 40 50 60 70 80 90 100 110 120 130 140 150 160 170 180 190 200
f1 (ppm)

(C)

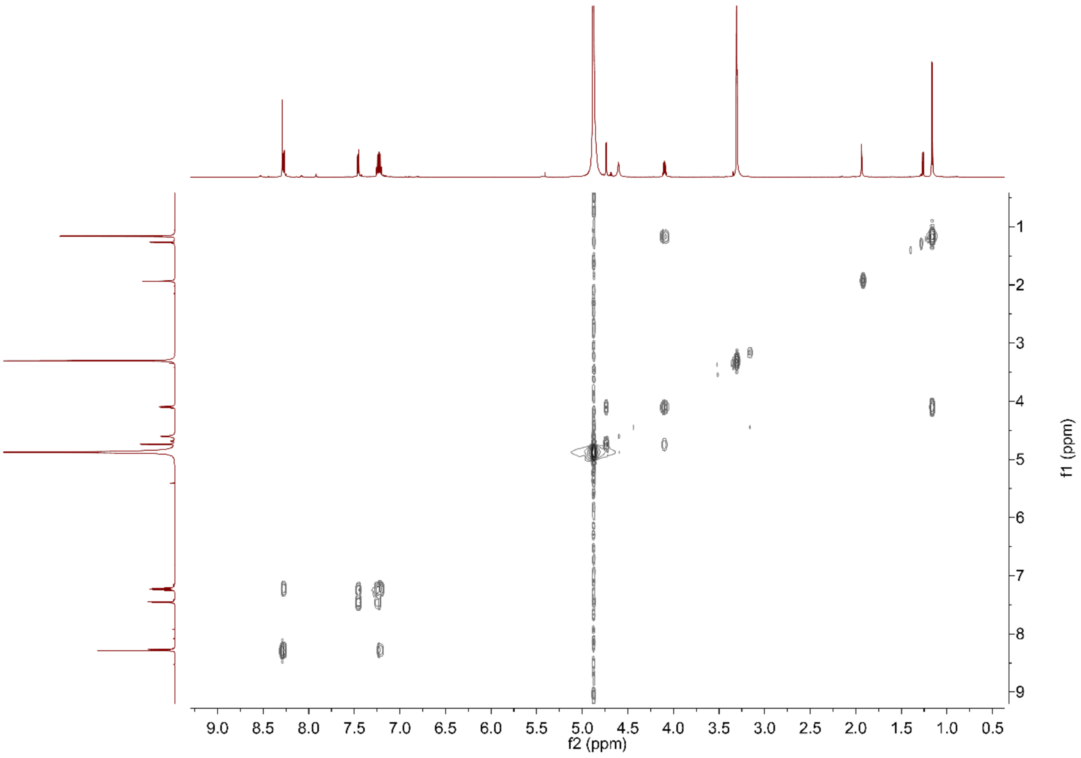

9.0 8.5 8.0 7.5 7.0 6.5 6.0 5.5 5.0 4.5 4.0 3.5 3.0 2.5 2.0 1.5 1.0 0.5
f2 (ppm)
1 2 3 4 5 6 7 8 9
f1 (ppm)

(D)

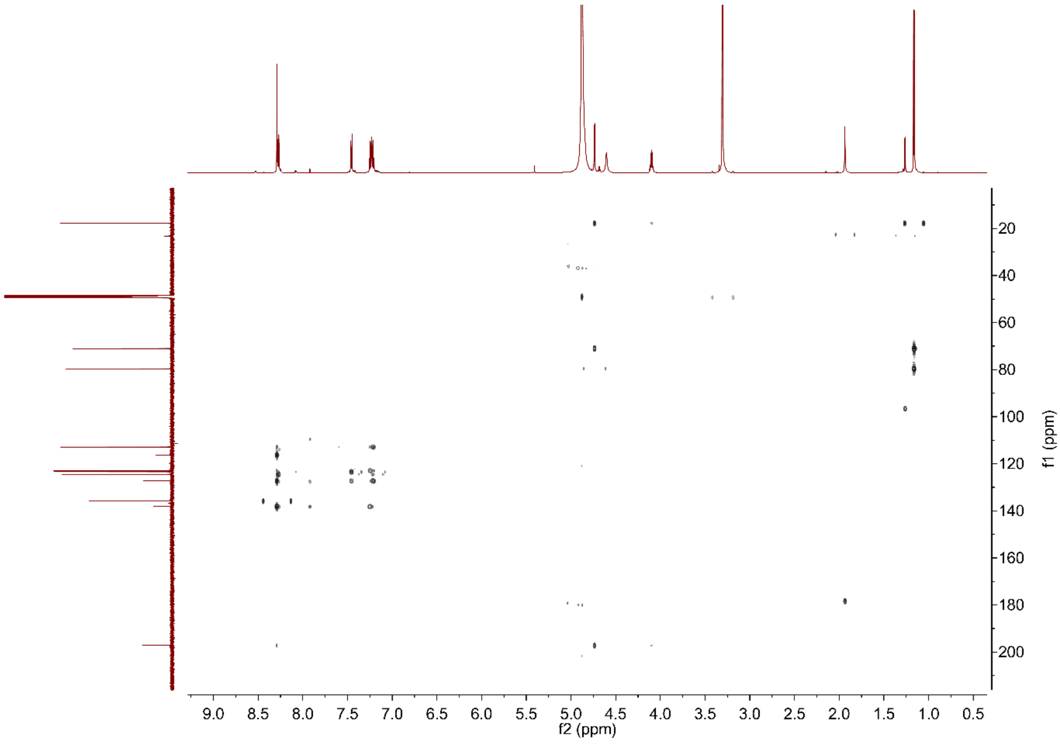

9.0 8.5 8.0 7.5 7.0 6.5 6.0 5.5 5.0 4.5 4.0 3.5 3.0 2.5 2.0 1.5 1.0 0.5
f2 (ppm)
20 40 60 80 100 120 140 160 180 200
f1 (ppm)

(E)

Sample Group **Info.**
Acquisition SW 6200 series TOF/6500 series
Version Q-TOF B.05.01 (B5125.2)

User Spectra

Fragmentor Voltage	Collision Energy	Ionization Mode
135	0	ESI

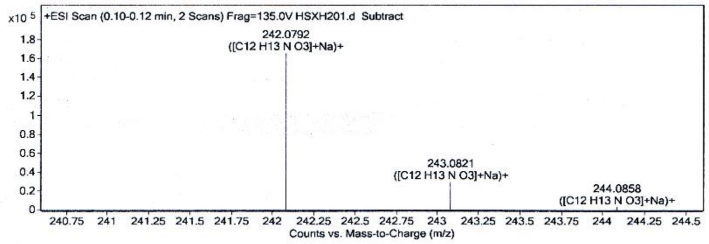

Peak List

m/z	z	Abund	Formula	Ion
242.0792	1	165883.44	C12 H13 N O3	(M+Na)+
243.0821	1	29232.97	C12 H13 N O3	(M+Na)+
245.1281	1	15575.36		
267.1572	1	20303.31		
437.1944	1	194185.81		
438.1971	1	57916.62		
453.1675	1	57000.45		
656.2836	1	118834.41		
657.2861	1	49291.63		
851.3963	1	18882.14		

Formula Calculator Element Limits

Element	Min	Max
C	3	120
H	0	240
O	0	60
N	0	3

Formula Calculator Results

Formula	CalculatedMass	CalculatedMz	Mz	Diff. (mDa)	Diff. (ppm)	DBE
C12 H13 N O3	219.0895	242.0788	242.0792	-0.40	-1.65	7.0000

(F)

Figure S5. (**A**)^{1}H NMR (600 MHz) spectrum of compound **1** in methanol-*d*$_4$; **(B)** ^{13}C NMR (600 MHz) spectrum of compound **1** in methanol-*d*$_4$; **(C)** ^{1}H-^{1}H COSY (600 MHz) spectrum of compound **1** in methanol-*d*$_4$; **(D)** HSQC (600 MHz) spectrum of compound **1** in methanol-*d*$_4$; (**E**)HMBC (600 MHz) spectrum of compound **1** in methanol-*d*$_4$; **(F)** HRESI spectrum of compound **1**.

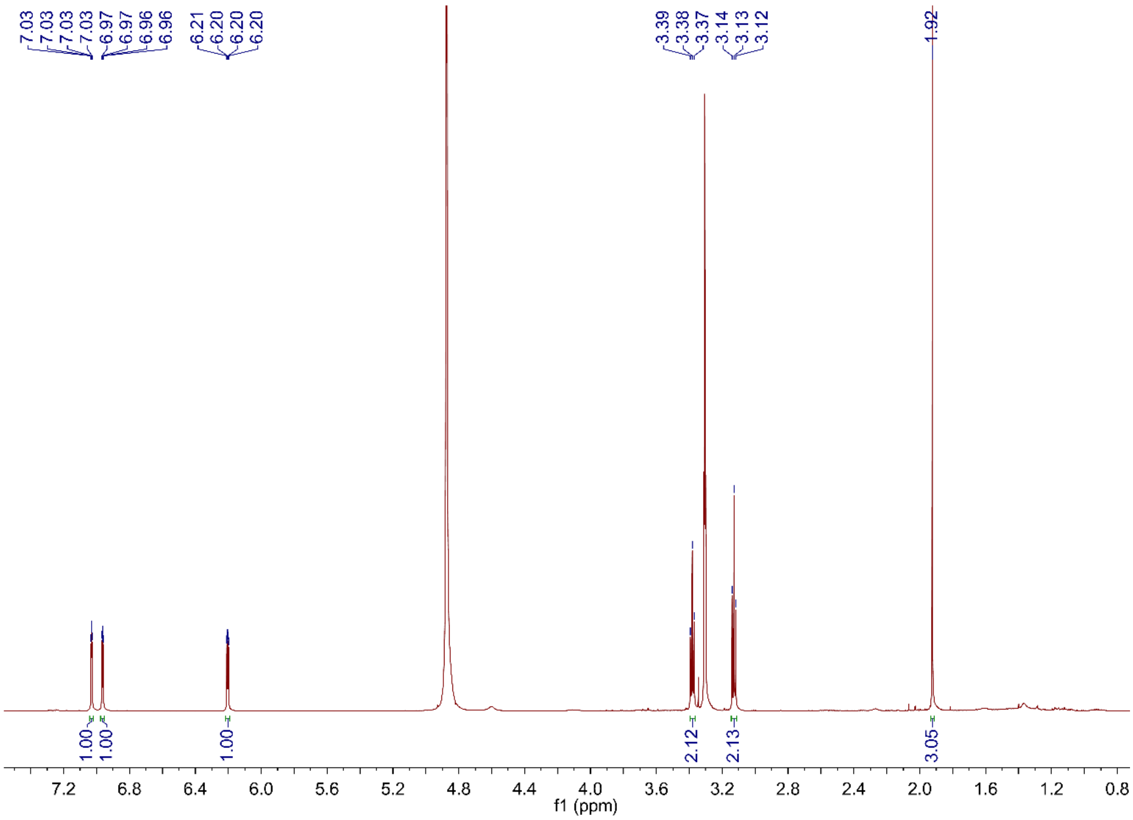
7.03
7.03
7.03
7.03
6.97
6.97
6.96
6.96
6.21
6.20
6.20
6.20
3.39
3.38
3.37
3.14
3.13
3.12
1.92
1.00
1.00
1.00
2.12
2.13
3.05
7.2
6.8
6.4
6.0
5.6
5.2
4.8
4.4
4.0
3.6
3.2
2.8
2.4
2.0
1.6
1.2
0.8
f1 (ppm)

(A)

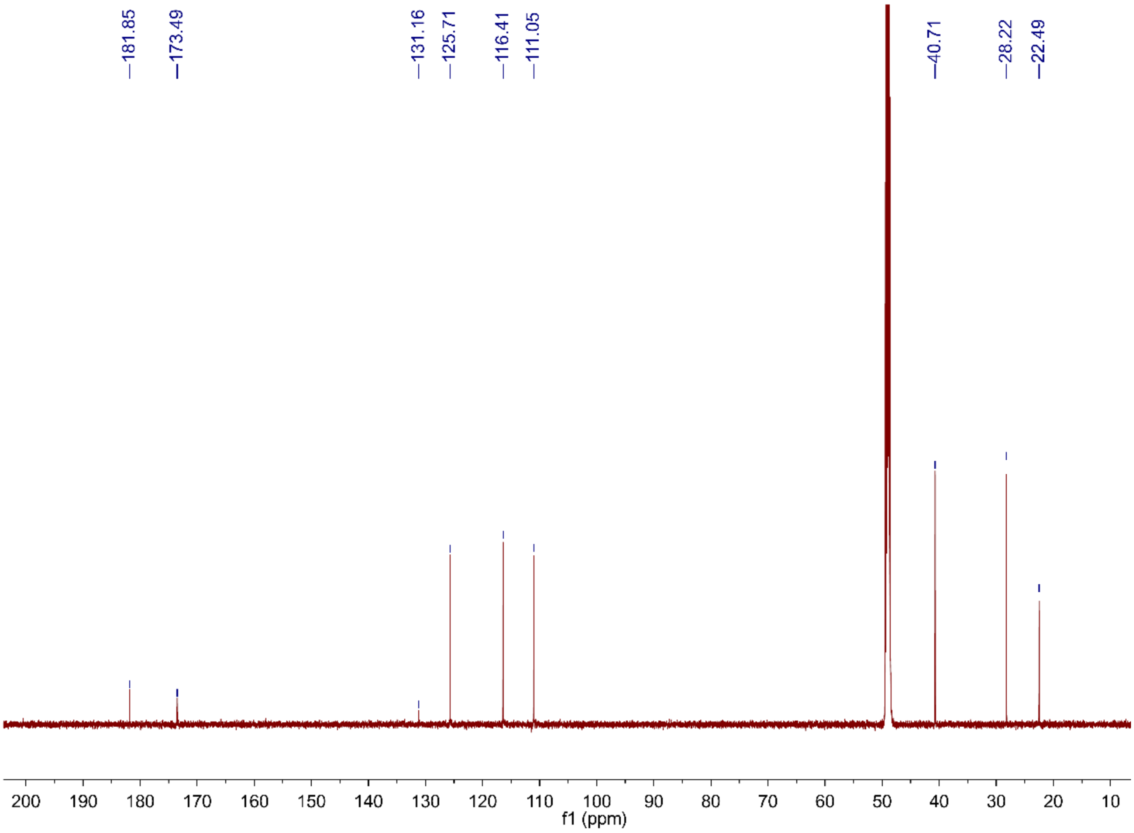
181.85
173.49
131.16
125.71
116.41
111.05
40.71
28.22
22.49
200
190
180
170
160
150
140
130
120
110
100
90
80
70
60
50
40
30
20
10
f1 (ppm)

(B)

Qualitative Analysis Report

**Data Filename**	hsx202.d	**Sample Name**	hsx202
**Sample Type**	Sample	**Position**	P1-A1
**Instrument Name**	Instrument 1	**User Name**	
**Acq Method**	s.m	**Acquired Time**	6/28/2019 2:19:59 PM
**IRM Calibration Status**	Success	**DA Method**	Default.m
**Comment**			

**Sample Group**		**Info.**
**Acquisition SW Version**	6200 series TOF/6500 series Q-TOF B.05.01 (B5125.2)	

User Spectra

Fragmentor Voltage	Collision Energy	Ionization Mode
135	0	ESI

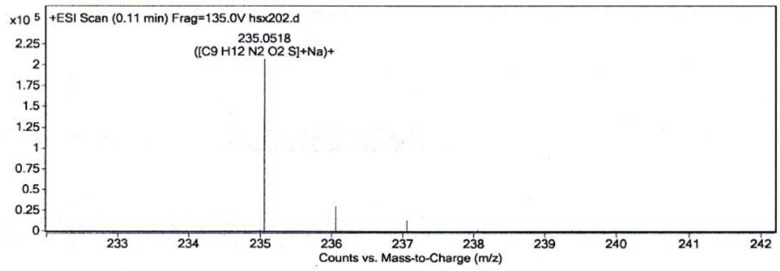

Peak List

m/z	z	Abund	Formula	Ion
64.016		23386.34		
121.0509	1	33690.73		
172.0943	1	30265.77		
213.0694	1	62232.98		
235.0518	1	207358.5	C9 H12 N2 O2 S	(M+Na)+
236.0542	1	29853.69	C9 H12 N2 O2 S	(M+Na)+
447.1132	1	100956.02		
448.116	1	24039.03		
922.0098	1	158389.8		
923.0114	1	30464.62		

Formula Calculator Element Limits

Element	Min	Max
C	3	60
H	0	120
O	0	30
S	0	5
N	0	5

Formula Calculator Results

Formula	CalculatedMass	CalculatedMz	Mz	Diff. (mDa)	Diff. (ppm)	DBE
C9 H12 N2 O2 S	212.0620	235.0512	235.0518	-0.60	-2.55	5.0000

--- End Of Report ---

Agilent Technologies Page 1 of 1 Printed at: 2:23 PM on: 6/28/2019

(C)

Figure S6. (**A**)^{1}H-NMR (600 MHz) spectrum of compound **2** in methanol-*d*4; (**B**)^{13}C-NMR (600 MHz) spectrum of compound 2 in methanol-*d*4; (**C**)HRESIMS spectrum of compound **2.**

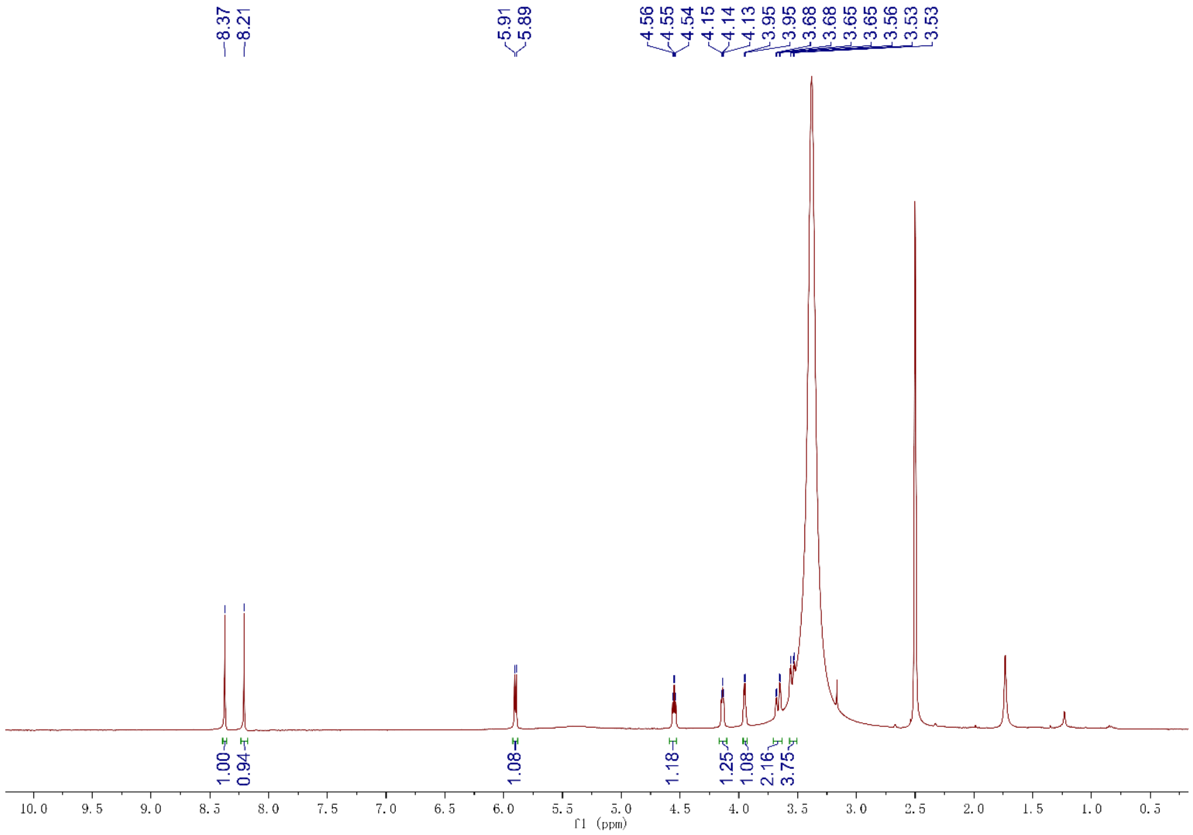

(A)

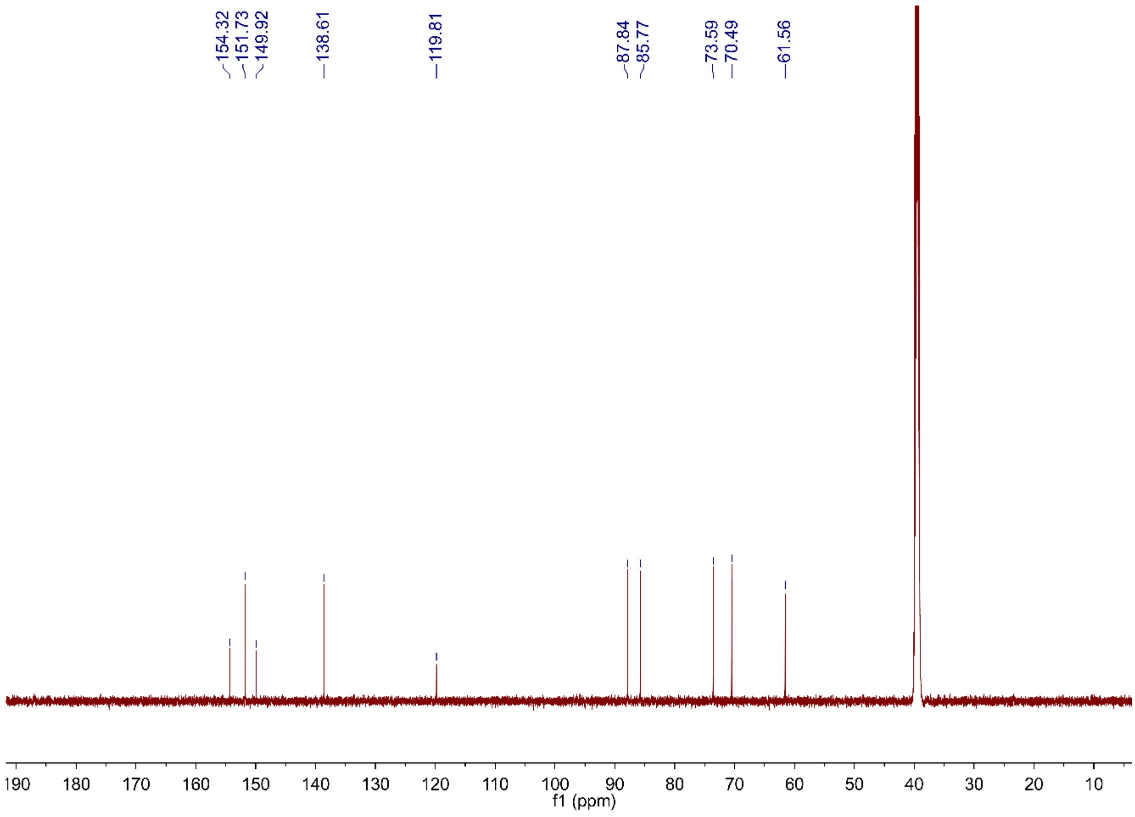

(B)

Figure S7. (**A**) ^{1}H-NMR (400 MHz) spectrum of compound **3** in DMSO-*d*$_6$; (**B**)^{13}C-NMR (150 MHz) spectrum of compound **3** in DMSO-*d*$_6$.

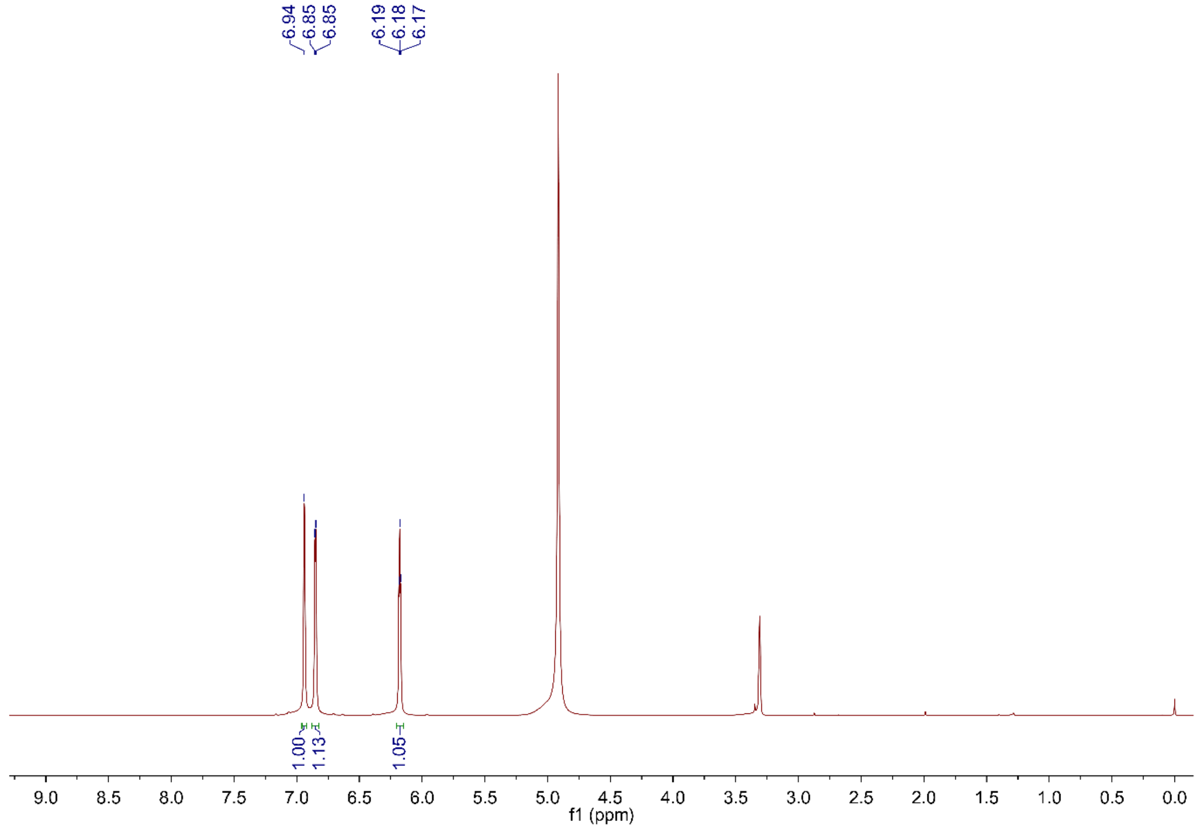

(A)

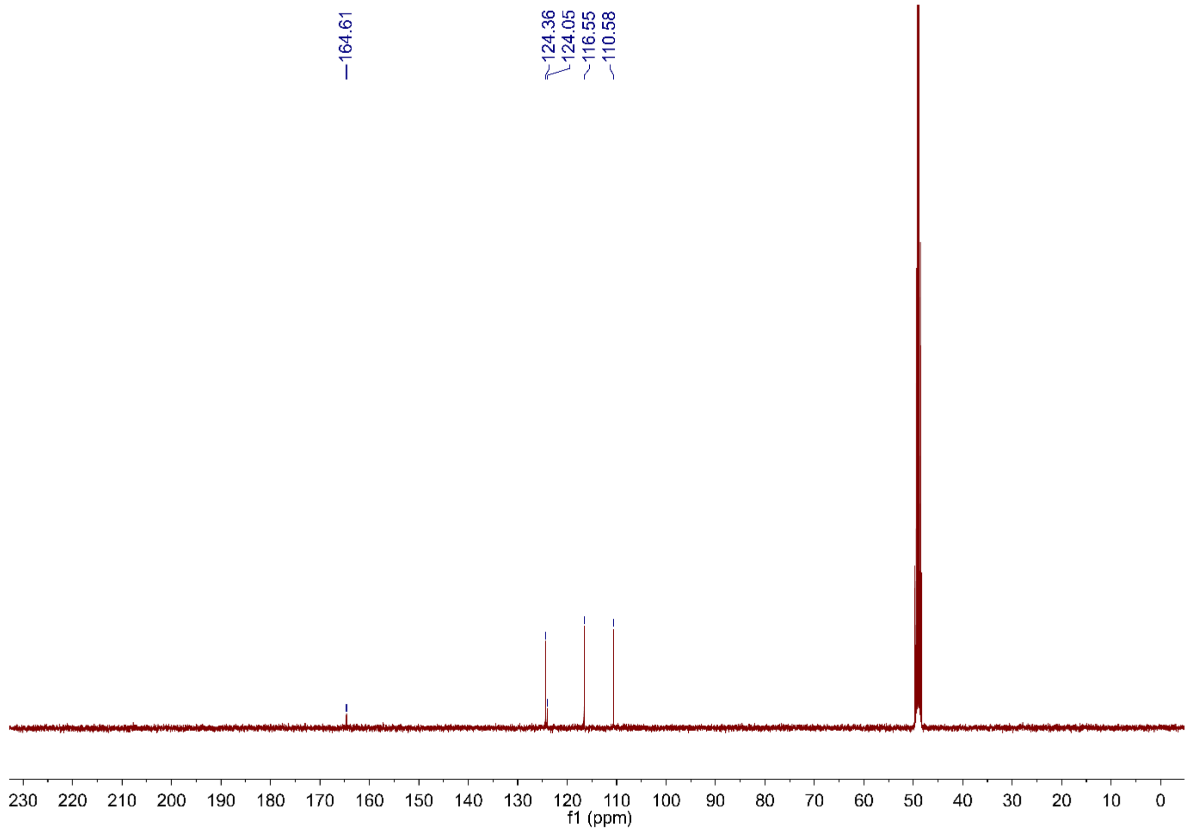

(B)

Figure S8. (**A**)^{1}H NMR (400 MHz) spectrum of compound **4** in methanol-*d*$_4$; (**B**) ^{13}C (100 MHz) spectrum of compound **4** in methanol-*d*$_4$.
